# Flavin-containing monooxygenase 3 as a potential player in diabetes-associated atherosclerosis

**DOI:** 10.1038/ncomms7498

**Published:** 2015-04-07

**Authors:** Ji Miao, Alisha V. Ling, Praveen V. Manthena, Mary E. Gearing, Mark J. Graham, Rosanne M. Crooke, Kevin J. Croce, Ryan M. Esquejo, Clary B. Clish, Morbid Obesity Study Group, Esther Torrecilla, Gumersindo Fernández Vázquez, Miguel A. Rubio, Lucio Cabrerizo, Ana Barabash, Andrés Sánchez Pernaute, Antonio J. Torres, David Vicent, Sudha B. Biddinger

**Affiliations:** 1 Division of Endocrinology, Boston Children’s Hospital, Harvard Medical School, Boston, Massachusetts, USA; 2 Isis Pharmaceuticals, Carlsbad, California, USA; 3 Cardiovascular Division, Brigham and Women’s Hospital, Harvard Medical School, Boston, Massachusetts, USA; 4 Metabolic Disease Program and Diabetes and Obesity Center, Sanford-Burnham Medical Research Institute, Orlando, Florida, USA; 5 Broad Institute, Cambridge, Massachusetts, USA; 6 Department of Endocrinology and Nutrition, Hospital Carlos III, Madrid 28029, Spain; 7 Instituto de Investigación Sanitaria del Hospital Universitario La Paz (IdiPAZ), Madrid 28046, Spain; 8 Department of Endocrinology and Nutrition, Hospital Clínico San Carlos, Madrid, 28040, Spain; 9 Medical School, Complutense University, Madrid, 28040, Spain; 10 Instituto de Investigación Sanitaria del Hospital Clínico San Carlos (IdiSSC), Madrid, 28040, Spain; 11 Department of Surgery. Hospital Clínico San Carlos, Madrid, 28040, Spain; 12 List of members and affiliations appears at the end of the paper.

## Abstract

Despite the well-documented association between insulin resistance and cardiovascular disease, the key targets of insulin relevant to the development of cardiovascular disease are not known. Here, using non-biased profiling methods, we identify the enzyme flavin-containing monooxygenase 3 (*Fmo3*) to be a target of insulin. FMO3 produces trimethylamine N-oxide (TMAO), which has recently been suggested to promote atherosclerosis in mice and humans. We show that *FMO3* is suppressed by insulin *in vitro*, increased in obese/insulin resistant male mice and increased in obese/insulin-resistant humans. Knockdown of FMO3 in insulin-resistant mice suppresses FoxO1, a central node for metabolic control, and entirely prevents the development of hyperglycaemia, hyperlipidemia and atherosclerosis. Taken together, these data indicate that FMO3 is required for FoxO1 expression and the development of metabolic dysfunction.

## Abstract (web summary)

The hepatic enzyme FMO3 has been linked to atherosclerosis. Here the authors show that FMO3 is upregulated in various models of diabetes and link FMO3 with key transcriptional regulators of hepatic glucose and cholesterol synthesis, thus proposing a mechanistic connection between diabetes and atherosclerosis.

## Graphical abstract

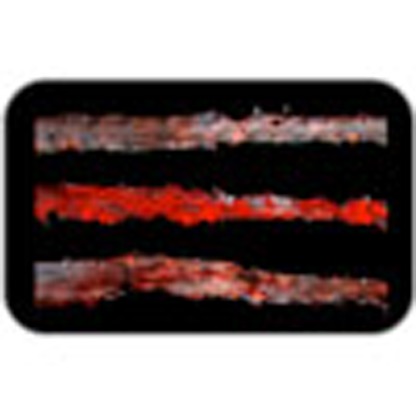

Obesity, metabolic syndrome and Type 2 diabetes are related disorders that have reached epidemic proportions in our society, with almost one in three adults meeting criteria for metabolic syndrome and 5–10% of the population with Type 2 diabetes. One of the major forms of morbidity and mortality associated with these disorders is cardiovascular disease (CVD). Diabetes increases the risk of cardiovascular disease by two- to four-fold and most individuals with diabetes ultimately die of cardiovascular disease^1^,^2^. Importantly, the risk of CVD in diabetic patients remains high even after optimal treatment with statin drugs^3^,^4^. This indicates the urgent need for developing better treatments to prevent CVD in diabetic patients.

Our ability to develop new therapies has been hampered by our lack of understanding of the specific mechanisms by which diabetes promotes CVD^5^. On the one hand, it is clear that defects in insulin action play a central role in the pathophysiology of diabetes^6^. However, insulin is a pleiotropic anabolic hormone, with many targets^6^. The goal of these studies was to identify novel targets of insulin action relevant to the development of diabetes-associated CVD.

## Results

### Transcriptional and metabolic profiling of LIRKO livers

To search for novel targets of insulin without the bias of an *a priori* hypothesis, we performed metabolic and transcriptional profiling on the livers of chow-fed male Liver Insulin Receptor Knockout (LIRKO) mice and their littermate Flox controls. The livers of LIRKO mice are unable to respond to insulin, allowing us to identify the targets of insulin action on the liver *in vivo*^7^. LIRKO mice show hyperglycaemia, hyperinsulinemia, and changes in lipid metabolism that are similar to individuals with mutations in the insulin receptor^8^,^9^,^10^. Moreover, they are markedly susceptible to atherosclerosis^11^.

**Figure 1:**
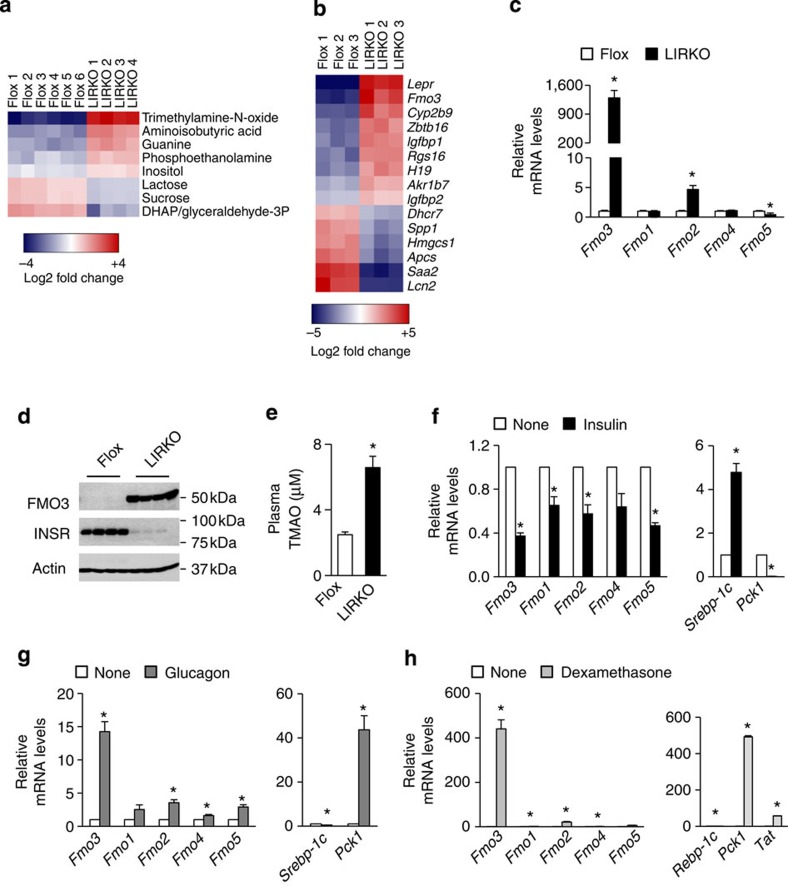
FMO3 is suppressed by insulin. (**a**–**e**) The livers of 2-month-old non-fasted male Flox and LIRKO mice were subjected to metabolic profiling (**a**) and microarray analysis (**b**); all metabolites and genes with an adjusted *P* value <0.05 are shown. Alternatively, hepatic gene expression (**c**) was measured using real-time PCR and protein levels (**d**) were measured by western blotting whole-cell lysates. Plasma TMAO levels (**e**) were measured using LC/MS. Data represent the mean and s.e.m.; *n*=5–11; *P<0.05 (Student’s *t*-test). (**f**–**h**) Gene expression was measured in primary hepatocytes from male rats (**f**,**g**) or mice (**h**) after 6 h of stimulation with insulin (**f**), glucagon (**g**) or dexamethasone (**h**). Average or representative results of two to four independent experiments are shown; *P<0.05 (Student’s *t*-test); *n*=3 replicates per condition.

Of the 175 metabolites profiled in the livers of LIRKO mice—including organic acids, bile acids, purines, pyrimidines and other compounds—the metabolite that showed the greatest fold change in LIRKO versus Flox livers was TMAO (Fig. 1a). In both humans and mice, TMAO concentrations correlate positively with CVD risk, and dietary supplementation with either TMAO or compounds that can be metabolized to TMAO increases atherosclerosis in mice^12^. In parallel, microarray profiling revealed that the second most highly upregulated transcript in LIRKO livers was *Fmo3*, the gene encoding the enzyme that produces TMAO^13^ (Fig. 1b). This was surprising, given that *Fmo3* had previously been shown to be regulated primarily by sex hormones and bile acids^13^. Taken together, these striking findings indicate that one of the most profound effects of insulin on the male mouse liver *in vivo* is to suppress *Fmo3* and TMAO.

Further studies in the livers of male LIRKO mice using real-time PCR analysis confirmed that *Fmo3* was increased >1,000-fold in the livers of LIRKO mice relative to their littermate Flox controls (Fig. 1c). In parallel, FMO3 protein was expressed robustly in LIRKO livers, but undetectable in Flox livers (Fig. 1d). Finally, TMAO levels in the plasma were elevated approximately 2.5-fold (Fig. 1e). Other *Fmo* genes were more modestly changed, with a three-fold increase in *Fmo2* and a 50% decrease in *Fmo5* (Fig. 1c).

### Insulin suppresses *Fmo3* in primary hepatocytes

To determine whether insulin could directly suppress *Fmo3*, we treated primary rat hepatocytes with insulin (Fig. 1f). Within 6 h of treatment, insulin suppressed the gluconeogenic enzyme phosphoenolpyruvate carboxykinase (*Pck1*) by >90%, and induced sterol regulatory element binding protein-1c (*Srebp-1c*) more than four-fold, as expected. In parallel, insulin suppressed *Fmo3* mRNA by 60%, and this was blunted by pharmacological inhibition of phosphatidylinositol (PI) 3-kinase (Supplementary Fig. 1).

Diabetes is associated not only with defects in insulin action, but also with multiple other changes in the hormonal milieu. In particular, there is increased action of glucagon and glucocorticoids, hormones that antagonize insulin action^14^,^15^,^16^,^17^. We found that glucagon increased *Fmo3* 14-fold, and dexamethasone (a synthetic glucocorticoid) increased *Fmo3* >400-fold (Fig. 1g,h). Similarly, *Pck1* was induced 40-fold by glucagon, and more than 400-fold by dexamethasone (Fig. 1g,h).

The other genes in the *Fmo* family showed similar, but more modest, responses to insulin, glucagon and dexamethasone. Insulin decreased expression of *Fmo1*, *Fmo2* and *Fmo5* by 35–50%, whereas glucagon increased them two- to three-fold (Fig. 1f,g). Dexamethasone increased *Fmo1* two-fold, *Fmo2* twenty-fold and *Fmo5* six-fold (Fig. 1h).

### FMO3 knockdown suppresses FoxO1 and improves glucose tolerance

To determine whether FMO3 might play a role in the development of the diabetic phenotype, we used second generation antisense oligonucleotides (ASO) to knockdown FMO3 in LIRKO mice. We studied three groups: Flox mice treated with a control ASO, LIRKO mice treated with a control ASO and LIRKO mice treated with an ASO against *Fmo3*. Each mouse was injected with 50 mg kg^−1^ body weight of ASO by intraperitoneal injection each week. This ASO was previously shown to reduce hepatic *Fmo3* expression by 90% and plasma TMAO by almost 50% (ref. 13).

**Figure 2:**
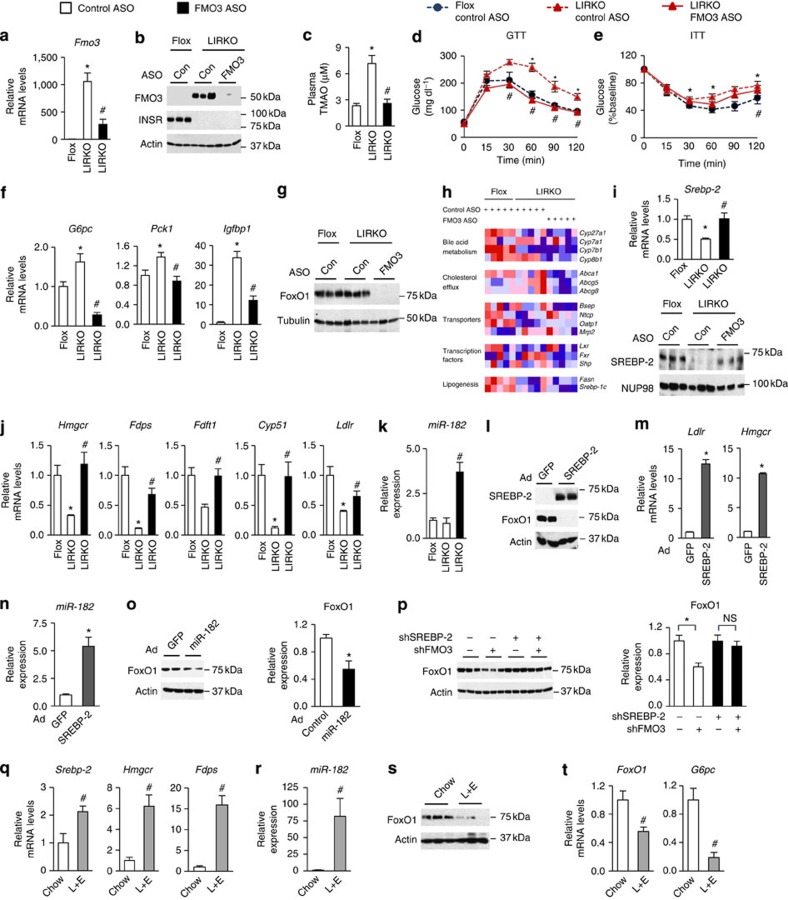
Knockdown of FMO3 suppresses FoxO1 via SREBP-2. (**a**–**k**) Four- to six-week-old male Flox and LIRKO mice were treated with control (Con) or FMO3 ASO for 7 weeks and killed in the non-fasted state. Hepatic gene expression (**a**,**f**,**h**,**i**,**j**,**k**) was measured by real-time PCR, and in (**h**) expressed as a heat map, with each column representing data from a single mouse. The data are row-normalized with red and blue representing high and low expression, respectively. Protein levels were measured by western blotting whole-cell lysates (**b**,**g**) or nuclear fractions (**i**). (**c**) TMAO was measured in plasma collected at the time of sacrifice using LC/MS. Glucose (**d**) and insulin (**e**) tolerance testing were performed after 5 weeks of ASO treatment. Data represent the mean and s.e.m.; *n*=5–7; *P<0.05 (Student’s *t*-test) LIRKO versus Flox mice treated with the control ASO; ^#^*P*<0.05 (Student’s *t*-test) control versus FMO3 ASO treatment of LIRKO mice. (**l**–**o**) Primary mouse hepatocytes were infected with control adenovirus or adenovirus expressing SREBP-2 (**l**–**n**) or miR-182 (**o**). Alternatively, shRNA expression plasmids were transfected into H2.35 hepatoma cells (**p**). Gene expression was measured by real-time PCR (**m**,**n**). Data represent mean and s.e.m.; *P<0.05 (Student’s *t*-test); *n*=3 wells per condition; results are representative of three independent experiments. (**l**,**o**,**p**) Whole-cell lysates were subjected to western blotting (**l**,**o**,**p**) and quantification (**o**,**p** right panels). (**o**,**p**) Data represent the mean and s.e.m. of three independent experiments; *P<0.05 (Student’s *t*-test). Representative images are shown in **l** and the left panels of **o**,**p**. (**q**–**t**) Eight- to ten-week-old male LIRKO mice were fed a chow diet with or without supplementation of lovastatin and ezetimibe (L+E) for 1 week and euthanized in the non-fasted state. Hepatic gene expression (**q**,**r**,**t**) was measured by real-time PCR. Hepatic FoxO1 protein (**s**) was measured by western blotting whole-cell lysates. Data represent the mean and s.e.m.; *n*=5–7; ^#^*P*<0.05 (Student’s *t*-test).

The FMO3 ASO decreased *Fmo3* mRNA by ∼75% in LIRKO mice and markedly reduced FMO3 protein (Fig. 2a,b); it also reduced *Fmo2* mRNA by 50% but it did not significantly change the other *Fmo* genes (Supplementary Fig. 2c). The FMO3 ASO did not alter body weight (Supplementary Fig. 2a), nor did it produce hepatic or renal toxicity (Supplementary Fig. 2b). As expected, FMO3 ASO treatment normalized plasma TMAO levels in LIRKO mice (Fig. 2c).

Surprisingly, knockdown of FMO3 completely normalized glucose tolerance and improved insulin tolerance in LIRKO mice (Fig. 2d,e). In parallel, FMO3 ASO significantly reduced expression of the gluconeogenic enzymes, glucose 6-phosphatase (*G6pc*) and *Pck1*, and insulin-like growth factor binding protein 1 (*Igfbp1*) (Fig. 2f).

*G6pc*, *Pck1* and *Igfbp1* are all targets of the transcription factor forkhead box O1 (FoxO1). FoxO1 drives gluconeogenic gene expression and is inhibited by insulin^18^,^19^. We found that FMO3 ASO treatment markedly reduced FoxO1 protein levels (Fig. 2g). The effects of FMO3 knockdown on FoxO1 were unlikely to be due to off-target effects of the ASO, as a second ASO targeting FMO3 also reduced FoxO1 protein in LIRKO livers (data not shown) and three additional shRNA constructs targeting FMO3 reduced FoxO1 protein levels in parallel with their ability to knockdown FMO3 *in vitro* (Supplementary Fig. 2g-i).

### Knockdown of FMO3 suppresses FoxO1 via SREBP-2

FMO3 is known primarily for its role in xenobiotic metabolism and more recently, cholesterol metabolism, via its product TMAO^20^. Though the specific molecular targets remain unclear, mice with dietary induced increases in TMAO have been shown to have altered cholesterol absorption, reverse cholesterol transport and bile acid metabolism^13^,^21^. We therefore examined the effects of FMO3 ASO treatment on a panel of genes involved in the uptake, synthesis and excretion of cholesterol (Fig. 2h). The bile acid synthetic enzyme *Cyp8b1*, which determines the bile salt profile and is known to be driven by FoxO1, was decreased by knockdown of FMO3. However, we did not observe consistent, significant effects of the FMO3 ASO on any of the following: the other bile acid enzymes (*Cyp7a1*, *Cyp7b1* or *Cyp27a1*); the cholesterol and bile transporters (*Abca1*, *Abcg5*, *Abcg8*, *Ntcp*, *Mrp2, Oatp1* or *Bsep*); the transcriptional regulators, *Lxr*, *Fxr* and *Shp*; or the lipogenic enzyme *Fasn* and its regulator *Srebp-1c*. Finally, *Klf15* and *Hmgcs2*, recently found to be reduced by FMO3 knockdown in other models^22^, trended downwards with FMO3 ASO treatment, but also did not consistently reach significance (Supplementary Fig. 2j).

On the other hand, we observed profound and consistent changes in the cholesterol synthetic enzymes and their regulator, sterol regulatory element binding protein- 2 (SREBP-2)^23^. SREBP-2 is induced by the depletion of cholesterol in the endoplasmic reticulum^24^. We have previously shown SREBP-2 to be decreased in LIRKO livers^25^. Interestingly, knockdown of FMO3 largely normalized expression of *Srebp-2* mRNA and the amounts of nuclear SREBP-2 protein (Fig. 2i). The SREBP-2 targets, 3-hydroxy-3-methylglutaryl-CoA reductase (*Hmgcr*), farnesyl diphosphate synthase (*Fdps*), squalene synthase (*Fdft1*), *Cyp51* and the LDL receptor (*Ldlr*) were also normalized in LIRKO mice by treatment with FMO3 ASO (Fig. 2j).

One potential link between FoxO1 and SREBP-2 is miR-182. This microRNA has been shown to directly target the 3′ UTR of FoxO1 and thereby suppress FoxO1 expression^26^,^27^,^28^. Importantly, miR-182 is encoded by a miRNA locus that is activated directly by SREBP-2 (ref. 29). We therefore measured miR-182 expression in the livers of LIRKO mice treated with FMO3 ASO. Consistent with the induction of SREBP-2 in these livers (Fig. 2i), miR-182 was increased four-fold (Fig. 2k).

To directly test the roles of SREBP-2 and miR-182 in the regulation of FoxO1, we overexpressed SREBP-2 via an adenovirus in primary mouse hepatocytes (Fig. 2l). Overexpression of SREBP-2 was sufficient to induce *Ldlr*, *Hmgcr* and miR-182 (Fig. 2m,n). In parallel, FoxO1 protein was suppressed (Fig. 2l). Overexpression of miR-182 also reduced FoxO1 protein, but the effects were more modest, suggesting that SREBP-2 may suppress FoxO1 via other mechanisms as well (Fig. 2o).

We also knocked down SREBP-2 in mouse hepatocyte-derived H2.35 cells using an shRNA construct. This construct reduced expression of *Srebp-2* and its targets, *Hmgcr*, *Ldlr* and miR-182 (Supplementary Fig. 2k). Moreover, knockdown of SREBP-2 abolished the effects of FMO3 knockdown on FoxO1 protein levels, indicating that SREBP-2 is required for the effects of FMO3 knockdown on FoxO1 (Fig. 2p; right panel shows quantification).

Taken together, these data suggest that knockdown of FMO3, perhaps by lowering endoplasmic reticulum cholesterol, induces SREBP-2 and thereby suppresses FoxO1. Consistent with this, knockdown of FMO3 reduced total hepatic cholesterol in the livers of LIRKO mice (Supplementary Fig. 2f), though this only reached significance in the presence of excess dietary cholesterol (Supplementary Fig. 3b).

To determine whether a reduction in hepatic cholesterol was sufficient to suppress FoxO1, we treated LIRKO mice with lovastatin and ezetimibe for 1 week^30^. This combination of drugs reduces hepatic cholesterol by inhibiting cholesterol synthesis and absorption^31^. As expected, lovastatin/ezetimibe treatment strongly induced *Srebp-2* and its targets in LIRKO livers^25^ (Fig. 2q). It also induced miR-182 (Fig. 2r) and suppressed *FoxO1* mRNA, FoxO1 protein and *G6pc* mRNA (Fig. 2s,t).

### FMO3 knockdown prevents hypercholesterolemia and atherosclerosis

**Figure 3:**
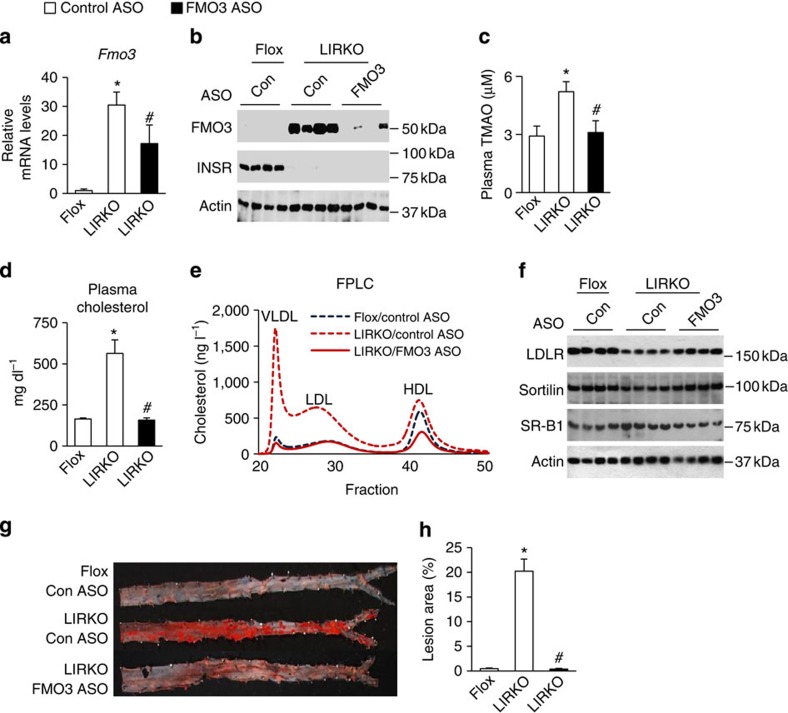
Knockdown of FMO3 prevents the development of atherosclerosis in LIRKO mice. At 4 to 6 weeks of age, male Flox and LIRKO mice were placed on an atherogenic Paigen diet, treated with control (Con) or FMO3 ASO for 16 weeks, and killed in the non-fasted state. Hepatic gene expression (**a**) was measured by real-time PCR. Hepatic protein levels (**b**,**f**) were measured by western blotting whole-cell lysates. (**c**,**e**) Plasma taken at the time of sacrifice was used to measure TMAO (**c**) or pooled (*n*=5–7 mice per group) and subjected to FPLC analysis (**e**). Total cholesterol (**d**) was measured after 12 weeks of ASO treatment and a 4-h fast. (**g**,**h**) Abdominal aortas were dissected and stained with Oil-Red-O (representative images shown in **g**). Lesion area was quantified and expressed as a percentage of the whole aorta (**h**). In the above *in vivo* experiments, data represent the mean and s.e.m.; *n*=5–13; *P<0.05 (Student’s *t*-test) LIRKO mice treated with the control ASO versus Flox mice; ^#^*P*<0.05 (Student’s *t*-test) control ASO versus FMO3 ASO-treated LIRKO mice.

We also asked whether knockdown of FMO3 could prevent atherosclerosis in LIRKO mice. Though LIRKO mice are not hypercholesterolemic relative to Flox mice on a chow diet (Supplementary Fig. 2e), they do develop hypercholesterolemia and atherosclerosis in response to the stress of the Paigen diet (15% fat, 1% cholesterol, 0.5% cholic acid)^11^. We therefore challenged the mice with the Paigen diet for 4 months. *Fmo3* mRNA was only 30-fold increased in LIRKO versus Flox livers on the Paigen diet, perhaps because of the presence of cholic acid, which induces *Fmo3* expression in normal mice^13^. Nonetheless, in LIRKO mice, treatment with ASO against FMO3 reduced *Fmo3* mRNA by ∼50%; more importantly, FMO3 protein and TMAO levels were normalized by FMO3 ASO treatment (Fig. 3a-c). Body weights were similar in all three groups, and *Srebp-2* and its targets were increased in LIRKO livers by the FMO3 ASO (Supplementary Fig. 3a,c).

In the presence of the Paigen diet, LIRKO mice developed severe hypercholesterolemia that was entirely prevented by the knockdown of FMO3 (Fig. 3d). Similar results were obtained on a Western diet (21% fat, 0.2% cholesterol, 34% sucrose), which lacks cholic acid (Supplementary Fig. 3d–g). In particular, knockdown of FMO3 decreased VLDL- and LDL-associated cholesterol in LIRKO mice (Fig. 3d,e). This was associated with an increase in LDL receptor, which removes atherogenic lipoproteins from the plasma, and sortilin, which both promotes clearance and inhibits secretion of atherogenic lipoproteins^32^ (Fig. 3f). SR-B1, which participates in reverse cholesterol transport by removing HDL cholesterol from the serum^33^, was decreased; however, HDL was also decreased, suggesting a possible discordance between SR-B1 protein levels and activity. In any case, consistent with the prevention of hypercholesterolemia, FMO3 knockdown completely prevented the development of atherosclerosis in Paigen-fed LIRKO mice (Fig. 3g,h).

### FMO3 is increased in other mouse models of diabetes

**Figure 4:**
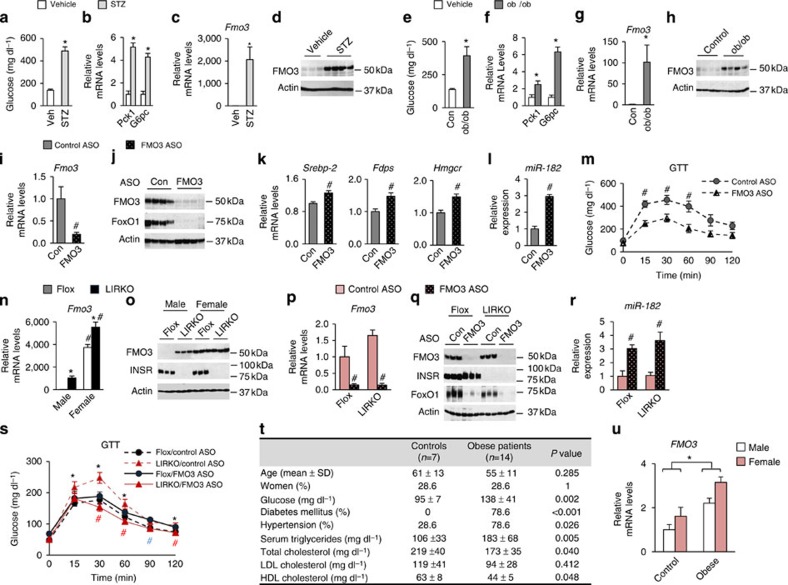
FMO3 in obese/diabetic mice and humans. (**a**–**h**) Eight- to ten–week-old male mice were killed in the non-fasted state. (**a**–**d**) Streptozotocin (STZ)-treated mice were compared with their vehicle-treated controls (Veh) and (**e**–**h**) *ob/ob* mice were compared with their wild-type controls (Con); all mice were killed in the non-fasted state. (**i**–**m**) Six-week-old male *ob/ob* mice were treated with control (Con) or FMO3 ASO for 4 weeks and were killed in the non-fasted state. Hepatic gene expression (**b**,**c**,**f**,**g**,**i**,**k**,**l**) was measured using real-time PCR, and protein levels (**d**,**h**,**j**) were measured by western blotting whole-cell lysates. Glucose tolerance testing (**m**) was performed after 3 weeks of ASO treatment. Data represent the mean and s.e.m.; *n*=5–8; *P<0.05 (Student’s *t*-test) vehicle versus STZ treated (**a** to **d**), control versus *ob/ob* mice (**e** to **h**), control ASO-treated *ob/ob* mice versus FMO3 ASO-treated *ob/ob* mice (**i** to **m**). (**n**–**o**) Two- to three-month-old male and female Flox and LIRKO mice were killed in the non-fasted state. Hepatic gene expression (**n**) was measured using real-time PCR, and protein levels (**o**) were measured by western blotting whole-cell lysates. (**p**–**s**) Four- to six-week-old female Flox and LIRKO mice were treated with control (Con) or FMO3 ASO for 7 weeks and killed in the non-fasted state. Hepatic gene expression (**p**,**r**) was measured by real-time PCR, and protein levels were measured by western blotting whole-cell lysates (**q**). Glucose tolerance testing (**s**) was performed after 3 weeks of ASO treatment. Data represent the mean and s.e.m.; *n*=5; *P<0.05 (Student’s *t*-test) Flox versus LIRKO mice with control ASO treatment; ^#^*P*<0.05 (Student’s *t*-test) control versus FMO3 ASO-treated mice of the same genotype (blue # is for Flox mice and red # is for LIRKO mice). (**t**,**u**) Liver biopsies were taken from age and sex-matched patients undergoing bariatric surgery (Obese) or other abdominal surgeries (Controls), and FMO3 expression was measured by real-time PCR (**u**); biochemical and physiological data from these patients are shown (**t**). Data represent the mean and s.e.m., as described in Methods. *P* values were determined by the Mann–Whitney test for continuous variables and by the *χ*^2^ test for categorical variables (**t**) or the Student’s *t*-test (**u**).

We also examined FMO3 in other models of diabetes. Streptozotocin (STZ)-treated mice are a model of Type 1 diabetes, as STZ treatment is toxic to the beta-cells of the pancreas, rendering STZ-treated mice insulin deficient. As expected, male STZ mice were hyperglycaemic with four- to five-fold increases in *Pck1* and *G6pc* expression in their livers relative to their untreated controls (Fig. 4a,b). *Fmo3* mRNA, however, was induced over 1,000-fold, and FMO3 protein was markedly induced (Fig. 4c,d).

Leptin-deficient *ob/ob* mice are a commonly used model of obesity/Type 2 diabetes. Male *ob/ob* mice were hyperglycaemic with increased hepatic expression of the gluconeogenic enzymes *Pck1* and *G6pc* relative to their lean controls (Fig. 4e,f). FMO3 mRNA and protein were also markedly increased in the livers of *ob/ob* mice (Fig. 4g,h). Blocking the effects of hyperinsulinemia in *ob/ob* mice by knocking down the insulin receptor further induced FMO3 protein, consistent with the notion that the signaling pathways utilized by insulin to suppress *Fmo3* become partially resistant to insulin in *ob/ob* mice (Supplementary Fig. 4a).

Treatment with the FMO3 ASO markedly reduced FMO3 in *ob/ob* livers (Fig. 4i,j). In parallel, it increased *Srebp-2*, the cholesterologenic enzymes, and miR-182 (Fig. 4k-l). Again, FoxO1 protein levels were lowered to near undetectable levels, and glucose tolerance was significantly improved (Fig. 4j,m). Thus, in *ob/ob* mice, as in LIRKO mice, the knockdown of FMO3 can activate SREBP-2/miR-182 and suppress FoxO1 and hyperglycaemia.

### FMO3 in female LIRKO mice

Taken together, these data indicate that FMO3 expression, which is directly inhibited by insulin and induced by glucagon and corticosteroids, is increased in male mice with insulin deficiency/insulin resistance. However, FMO3 expression is sexually dimorphic, with regulation at the levels of transcription, translation/protein stability and enzyme activity^13^. Thus, FMO3 in females livers is >1,000-fold higher at the mRNA level and three-fold higher at the enzyme activity level^13^.

We therefore examined the expression of FMO3 in LIRKO females. Flox females had 4,000 times more *Fmo3* mRNA and markedly more FMO3 protein than their male counterparts, as expected (Fig. 4n,o). Though knockout of the insulin receptor increased *Fmo3* expression, the effects were modest and only reached significance in some cohorts. To determine whether FMO3 still regulated FoxO1 in female mice, we knocked down FMO3 in both Flox and LIRKO female mice (Fig. 4p–s). Indeed, FMO3 knockdown increased expression of miR-182 (Fig. 4r) and suppressed FoxO1 (Fig. 4q) and its targets (Supplementary Fig. 4b) in females, just as it did in males. In LIRKO mice, this resulted in improved pyruvate tolerance (Supplementary Fig. 4c), which reflects gluconeogenic capacity, and improved glucose tolerance (Fig. 4s). In Flox mice, the reduction of FoxO1 protein was not associated with an improvement in glucose tolerance, consistent with previous studies showing that the effects of FoxO1 knockout on glucose metabolism are manifested primarily in the insulin-resistant state^34^.

The fact that FMO3 knockdown had similar effects in males and females suggests that the FMO3/TMAO pathway is important in metabolic control in both sexes, though its regulation is different. Such extreme sexual dimorphism of FMO3 is only observed in certain species of mice, such as *Mus musculus* and *Mus domesticus*^35^. Even other rodent species, such as the mouse strain *Mus caroli* and *Rattus norvegicus*, do not show sexually dimorphic expression of FMO3 in their livers^35^. In humans, the sex effect on TMAO/FMO3 is absent or much more modest, as TMAO levels are not significantly different between males and females^21^, and hepatic *Fmo3* mRNA is increased only 50% to three-fold in females^13^.

### *FMO3* is increased in obese/insulin resistant subjects

To begin to explore the relationship between FMO3 and diabetes in humans, we examined *FMO3* expression in the livers of age and sex-matched patients undergoing either bariatric surgery (Obese) or other abdominal surgeries (Controls). All of the individuals undergoing bariatric surgery had a BMI>33.0 (with a mean BMI of 41.8), and 79% were diabetic. Consistent with their metabolic derangements, the obese group had higher levels of serum triglycerides and lower HDL than the controls (Fig. 4t). *FMO3* was significantly increased in the morbidly obese group (Fig. 4u). However, the effect was more modest than seen in the mouse models, possibly because the diabetic patients were treated with insulin and/or other medications to improve insulin sensitivity, and because the liver biopsies were taken under the fasting conditions of surgery.

## Discussion

Over the past 4 years, FMO3 and TMAO have emerged as key components of a complex axis integrating diet and the gut microbiome with atherosclerosis^12^,^13^,^21^,^36^, and knockdown of FMO3 was recently shown in mouse models of hyperlipidemia to prevent atherosclerosis and improve the metabolic phenotype^37^. Here, we have independently identified FMO3 and TMAO by performing non-biased profiling in male mice with diabetes-associated atherosclerosis. Importantly, we find that FMO3 is required for expression of FoxO1, a key node within the cell, controlling growth, differentiation and metabolism^19^,^38^. In addition to regulating glucose production, FoxO1 regulates bile acid, lipoprotein and fatty acid metabolism^34^,^39^,^40^,^41^,^42^,^43^,^44^,^45^,^46^. It is clear that knockdown of FoxO1 has profound effects, reversing most, but not all aspects of the insulin-resistant state^34^,^39^.

In female mice, FMO3 is not strongly induced by insulin resistance and non-diabetic female mice have high levels of FMO3/TMAO^21^, but do not necessarily manifest hyperglycaemia or atherosclerosis. In male mice, FMO3 is strongly induced by diabetes, but transient overexpression of FMO3 in lean male mice does not produce hyperglycaemia (data not shown). In both male and female mice, FMO3 is required for the development of the diabetic phenotype. Taken together, these data suggest that FMO3, which is differentially regulated in male and female mice, is necessary but not sufficient for the development of the diabetic phenotype.

Several lines of evidence support the notion that the link between diabetes and FMO3/TMAO will be important in humans. First, our data in human liver samples show that FMO3 is increased in the livers of obese/insulin-resistant individuals undergoing gastric bypass. Second, previous studies have shown that increased plasma levels of TMAO are associated with increased levels of serum glucose and diabetes in patients undergoing elective coronary angiography^36^. Finally, using Meta-Analysis of Glucose and Insulin-related traits Consortium data set (*n*=15,234 nondiabetic individuals), we found several SNPs in the FMO3 locus to be associated with blood glucose levels (*P*=4 × 10^−5^)^47^, though they did not reach genome-wide significance (Supplementary Fig. 4d).

The results of this work suggest that therapies to reduce FMO3/TMAO to normal levels may be particularly helpful in the prevention of diabetes-associated CVD. This may be through pharmacological manipulations to reduce FMO3 activity in diabetic patients, or by dietary interventions, such as the restriction of carnitine and/or choline, which serve as precursors of TMAO.

## Methods

### Animal, diets and treatments

Animals were housed in a twelve-hour light/dark cycle (0700–1900 hours). Unless otherwise indicated, mice were given standard chow and water *ad libitum*, and killed in the non-fasted state, at 1400 hours. The type and length of the diet other than chow were indicated in the main text and figure legends. The Western diet (TD88137) and Paigen diets (TD09237) were obtained from Harlan Teklad. All procedures were approved by the Institutional Animal Care and Research Advisory Committee at Boston Children's Hospital.

LIRKO (Cre^+/−^, IR ^lox/lox^) mice^7^ and their littermate flox controls (Cre^−/−^, IR ^lox/lox^) were maintained on a mixed genetic background including 129/sv, C57BL/6, FVB and DBA. Male *ob/ob* mice and their lean, wild-type C57BL/6J controls were purchased from Jackson Laboratories.

For antisense oligonucleotides (ASO) experiments, four- to six-week-old mice were administered chemically modified ASO (50 mg kg^−1^ body weight) by intraperitoneal injection weekly for 7 weeks, or 16 weeks in the case of the atherosclerosis studies. Mice were killed 1 day after the final dose. Control (ISIS-141923, 5′-CCTTCCCTGAAGGTTCCTCC-3′), FMO3-specific (ISIS-555847, 5′-TGGAAGCATTTGCCTTTAAA-3′) and insulin receptor-specific (ISIS 401145 5′-GTGTTCATCATAGGTCCGTT-3′) chimeric 20-mer phosphorothioate oligonucleotides containing 2’O-methoxyethyl groups at positions 1 to 5 and 16 to 20 were diluted in normal saline before injection. Glucose tolerance test (GTT) and insulin tolerance tests (ITT) were performed after 5 weeks of ASO administration, unless otherwise indicated. For STZ treatment, eight- to nine-week-old male C57BL/6J mice were injected with STZ (180 mg kg^−1^ body weight) or vehicle (0.1 M citric acid, pH=4.2), and killed 7 days later. For the lovastatin/ezetimibe treatments, eight- to ten-week-old male mice were given free access to powdered chow with or without supplementation of 0.1% lovastatin and 0.025% ezetimibe (both w/w), and killed 7 days later.

### Glucose tolerance test, insulin tolerance test and pyruvate tolerance test

Mice were fasted (∼16 h for GTT and pyruvate tolerance test and 4 h for ITT) and then were intraperitoneally injected with 1 g kg^−1^ body weight glucose (GTT), 1 U kg^−1^ body weight insulin (ITT) or 2 g kg^−1^ body weight of sodium pyruvate (PTT)^8^,^48^.

### Phenotypic and biochemical characterization

Blood glucose was measured with a glucometer using whole blood from tail bleeds. Plasma total cholesterol (Thermo Scientific) and total triglycerides (Thermo Scientific) were measured using colorimetric assays per manufacturers’ instruction. Alternatively, livers were homogenized in 50 mM NaCl and lipid was extracted with chloroform and methanol (2:1) followed by a colorimetric assay per manufacturers’ instruction^25^.

Plasma metabolites were extracted with methanol followed by LC/MS analysis. TMAO *m*/*z*=76 peaks were monitored and areas under the curve were calculated^49^. Fast protein liquid chromatography (FPLC) Analysis^50^,^51^ was performed by the Lipid, Lipoprotein and Atherosclerosis Analysis Core Laboratory at Wake Forest University.

### Microarray and metabolomics analysis

Total RNA (RNeasy, Qiagen) was isolated from the livers of non-fasted male mice (RNA from two to three mice were pooled for each chip) and hybridized to Affymetrix MG-U74A-v2 chips per manufacturers’ instructions. Raw data were processed in R (www.r-project.org) using the open-source Bioconductor packages, *affy*^52^ and *limma*^53^. Samples were background corrected and normalized using robust multichip averaging (RMA)^52^. Adjusted *P*-values were calculated with the limma package by applying the Benjamini–Hochberg correction. Alternatively, metabolomics was performed^49^,^54^ on individual livers harvested from male Flox and LIRKO mice; *P* value was adjusted for multiple comparisons using a Bonferroni correction. Heat maps were generated in Excel for significantly altered genes or metabolites (adjusted *P*-value<0.05) and were coloured by log_2_ fold changes.

### Gene expression analysis

Gene expression was measured using real-time PCR. Total RNA was isolated by Trizol (Life technologies) or RNeasy kit (Qiagen). cDNA was synthesized by a reverse transcription kit (Applied Biosystems). The resultant cDNA was diluted Five- to ten-fold and used for real-time PCR analysis with SYBR green reagents (Thermo Scientific) in Applied Biosystems 7900 HT or 7000 instruments. Results were normalized to the house keeping genes 18S (*in vivo* studies) or 36B4 (*in vitro* studies). In some cases, gene expression data, after normalization to 18S, were expressed as a heat map made using GenePattern^55^. Primer sequences are listed in Supplementary Table 1. The value of the control group was set to 1, but actual Ct values for QPCR are given in Supplementary Table 2.

Alternatively, for quantification miR-182 (mmu-miR-182), Taqman assays (Applied Biosystems) were performed on RNA samples prepared using Trizol and the expression was normalized to housekeeper U6 snRNA. The value of the control group was set to 1.

### Construction of plasmids

Full length of mouse *Fmo3* cDNA was cloned into pcDNA3 vector by introducing KpnI and NotI restriction enzyme sites using PCR. Control shRNA against LacZ (5′-GTTCACGGCGACAACTGC-3′), shRNA against mouse FMO3 (#1: 5′-GCATTTACCAATCGGTCTTCA-3′; #2 5′-GCTGGGCAGCACAAGTAATAA-3′; #35′-GCTTCCACAGCAGGGACTATA-3′) and shRNA against SREBP-2 (5′-GGACAACACACAATATCATTG-3′) were constructed using the Block-it U6 system (Life Technologies).

### Adenovirus

Control adenovirus and adenovirus expressing human SREBP-2 were purchased (Eton Biosciences) and amplified in 293A cells (Life Technologies). Adenovirus expressing the precursor form of mmu-miR-182 was generated using the AdEasy XL Adenoviral Vector System. The DNA sequence from 157-bp upstream through 224-bp downstream of the mouse pri-miR-182 sequence was amplified by PCR with primers containing NheI and XhoI restriction sites on either end. This fragment was cloned into the pGEM-T vector (Promega), sequenced, transferred into the pShuttle vector and subsequently cloned into the pAd-Easy-1 vector (Agilent).

### Cell culture

Cells were maintained at 37 °C in a 5% CO_2_ mammalian cell culture incubator. H2.35 mouse hepatoma cells (ATCC) and 293A cells were maintained in DMEM media (25 mM glucose, Life Technologies) containing penicillin-streptomycin and 10% fetal bovine serum. Cells were tested every 4 months to ensure no mycoplasma contamination. Transient transfection was performed using lipofectamine 2000 (Life Technologies) when cells reach 70% confluency according to a protocol suggested by the manufacturer. Cells were then harvested for protein or RNA extraction 48 h post transfection.

### Primary hepatocyte studies

Primary rat hepatocytes were isolated from 8-week-old male Sprague–Dawley rats (Harlan)^56^. After isolation, cells were suspended in William’s E medium (Life Technologies) containing penicillin-streptomycin, 100 nM glutamine (medium A) and 10% fetal bovine serum and 1 × 10^6^ rat hepatocytes were placed on rat tail collagen I (BD Biosciences)-coated six-well plates. Four hours later, cells were washed twice with PBS and incubated overnight in medium A supplemented with 100 nM dexamethasone, 100 nM triiodothyronine and 1 nM insulin (fasting medium). Cells were then washed twice with PBS and incubated for 6 h in either medium A containing 100 nM dexamethasone and 100 nM triiodothyronine with or without 100 nM insulin supplementation, or medium A containing 100 nM dexamethasone with or without 100 nM glucagon supplementation.

Similarly, male mouse hepatocytes were isolated, plated at a density of 0.5 × 10^6^ cells per well. Cells were incubated overnight in medium A without dexamethasone, washed and then incubated for 6 h with either vehicle (DMSO) or 100 nM dexamethasone.

For adenoviral infection, rat hepatocytes were infected with 10 to 50 MOI adenovirus 4 h after cells were placed on plates. Twenty-four hours later, cell media was replaced with fresh media. Cells then were collected 48 h post viral infection.

### Western blotting

Nuclear and cytoplasmic extracts were prepared by using a commercial kit (Thermo Scientific) per the manufacturer’s instruction. Alternatively, whole-cell lysates were prepared from the liver^25^, primary mouse hepatocytes^8^ and H2.35 cells^8^. Ten to fifty micrograms of lysates was loaded onto sodium dodecyl sulfate–PAGE (SDS–PAGE) gels and transferred onto a PVDF membrane (Thermo Scientific). After blocking in SuperBlock buffer (Thermo Scientific), blots were incubated overnight with a primary antibody (1:1,000 to 2,000 dilution). The secondary antibody conjugated with horseradish peroxidase (Thermo Scientific) and chemiluminescent ECL reagents (Thermo Scientific) were used to develop blots. ImageJ (NIH) was used to quantify densitometry of the bands on film. The LDLR antibody was a kind gift from Dr Alan Attie^57^. Other antibodies used in study were obtained from commercial sources and are listed in Supplementary Table 3. Noncropped western blot images are shown in Supplementary Fig. 5.

### Atherosclerosis

Animals were perfused with saline. The abdominal aortas were dissected free of adventitial fat, fixed with formalin and stained with Oil-Red-O. Aortas were then open longitudinally and pinned onto surfaces of black wax. Images of stained aortas were taken with a digital camera and lesion areas were quantified using computer-aided software (Image-Pro).

### Human studies

Human studies were approved by the Ethics Committee of the Hospital Clínico San Carlos, and all subjects gave informed consent. Liver biopsies were obtained from morbidly obese patients undergoing bariatric surgery or control patients undergoing surgery for gastroesophageal reflux disease (four patients), achalasia (one patient) and cholelithiasis (two patients), during the years 2004–2009 at the Hospital Clínico San Carlos. The bariatric surgery patients and controls were *post-hoc* age and sex matched. Clinical and biochemical data taken at the time of surgery were obtained from the chart. Clinical and biochemical data were available for all patients undergoing bariatric surgery. For the controls, glucose and total cholesterol levels were available in six patients, triglycerides in five patients, and HDL and LDL in three patients. For gene expression, RNA was isolated (TRI Reagent, Sigma-Aldrich), cDNA was synthesized (High-Capacity cDNA Reverse Transcription Kit, Applied Biosystems), and FMO3 gene expression was measured (TaqMan human FMO3 Gene Expression Assay Hs00199368_m1 and human 18 s rRNA as reference gene, Applied Biosystems). Expression was calculated via the ΔΔCt method.

### Statistical analysis

*Human studies*. For clinical parameters, *P* value was determined by Mann–Whitney test for continuous variables and by χ^2^ test for categorical variables. For gene expression studies, the expression values were not normally distributed. Therefore, a log transformation was performed after which the mean and s.e.m. were calculated, and a *t*-test was performed. Data are presented as the back-transformed values of the mean and s.e.m.

### *Mouse studies*

Sample sizes were based on standard lab protocols and previous studies, rather than power calculations, as the effect sizes were not known *a priori*. Animals were randomized to control and experimental groups. Atherosclerosis was measured in a blinded manner. Animals whose body weights were two or more s.d. values lower than the average of their groups were excluded (one animal in the Flox control ASO group in Fig. 3). Differences between groups were assessed by a two-tailed unequal variance Student’s *t*-test. Bars and error bars correspond to the mean and s.e.m., respectively. Representative results of two to four independent experiments are shown.

### In vitro studies

Gene expression studies were performed with triplicate wells and immunoblotting experiments were performed using duplicate wells. Average or representative results of two to five independent experiments are shown.

## Additional information

**Accession codes**: Microarray data have deposited in GEO under accession code GSE65624.

**How to cite this article:** Miao, J. *et al.* Flavin-containing monooxygenase 3 as a potential player in diabetes-associated atherosclerosis. *Nat. Commun.* 6:6498 doi: 10.1038/ncomms7498 (2015).

## Supplementary Material

Supplementary Information Supplementary Figures 1-5, Supplementary Tables 1-3 and Supplementary References
