# Supplementary material for: Flavin-containing monooxygenase 3 as a potential player in diabetes-associated atherosclerosis
Source: Nat Commun. 2015 Apr 7;6:6498. doi: 10.1038/ncomms7498 (PMC4391288; doi:10.1038/ncomms7498)
Supplement: Supplementary Information — Supplementary Figures 1-5, Supplementary Tables 1-3 and Supplementary References [file ncomms7498-s1.pdf]

Supplementary Information

Supplementary Figure 1

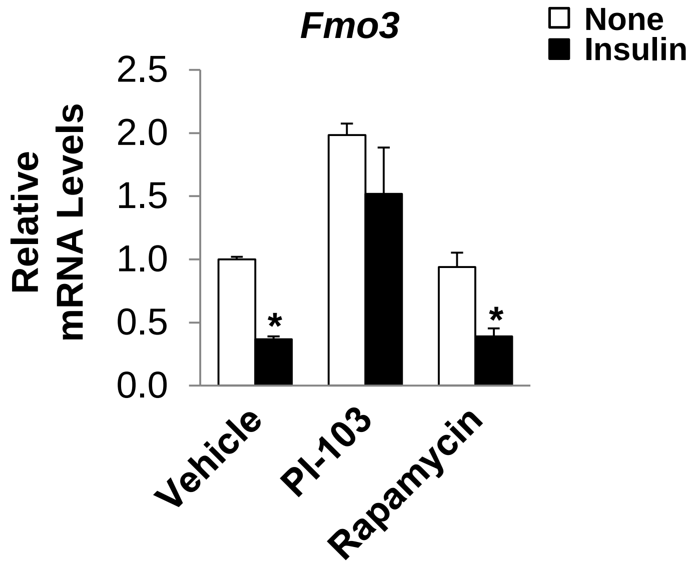

Supplementary Figure 1. ***In vitro* regulation of FMO3 in primary rat hepatocytes.** Primary rat hepatocytes were pre-treated with PI-103, an inhibitor of PI 3-kinase or rapamycin, an inhibitor of the mTORC1 complex, for 30 minutes prior to insulin stimulation. Data represent the mean ± SEM of triplicate wells; representative results of three independent experiments are shown. * $p < 0.05$ (Student's t-test) insulin treated versus untreated or vehicle treated cells.

Supplementary Figure 2

☐ Control ASO
■ FMO3 ASO

a

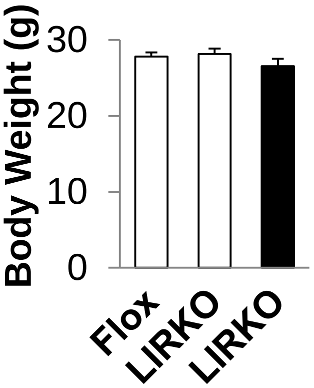

b

	ALT (IU L^{-1})	AST (IU L^{-1})	Albumin (mg dL^{-1})	Total Bilirubin (mg dL^{-1})	BUN (mg dL^{-1})	Creatinine (mg dL^{-1})
**Flox Control ASO**	20.25 ± 2.17	44.25 ± 1.75	2.09 ± 0.13	0.0675 ± 0.009	30.28 ± 2.76	0.0225 ± 0.0075
**LIRKO Control ASO**	63.40 ± 7.25*	236.40 ± 21.18*	1.84 ± 0.13*	0.132 ± 0.017*	40.84 ± 0.92	0.070 ± 0.0158*
**LIRKO FMO3 ASO**	63.75 ± 8.74	169.50 ± 21.18	1.65 ± 0.07	0.090 ± 0.016	41.70 ± 1.75	0.0325 ± 0.0075

c

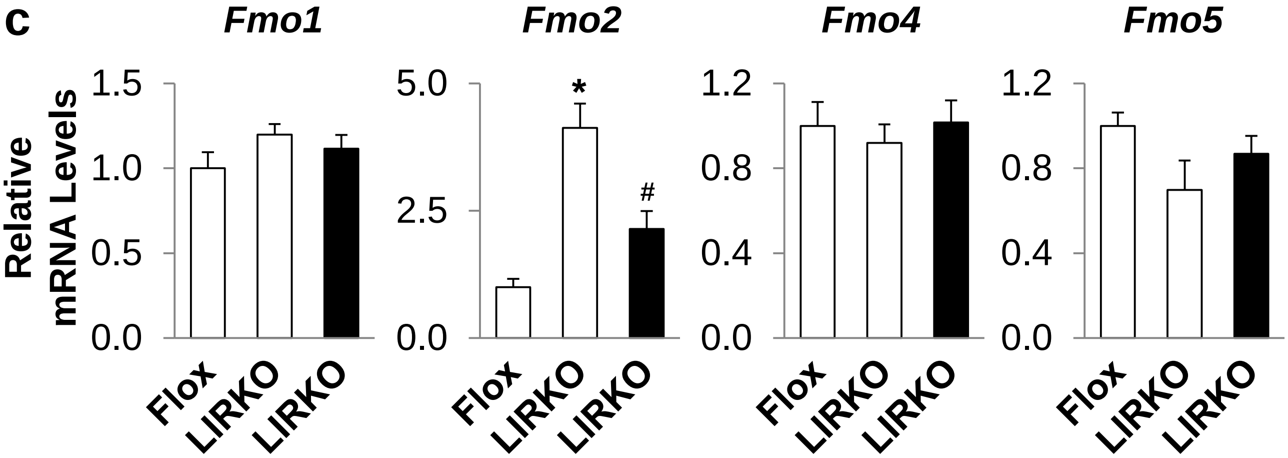

d

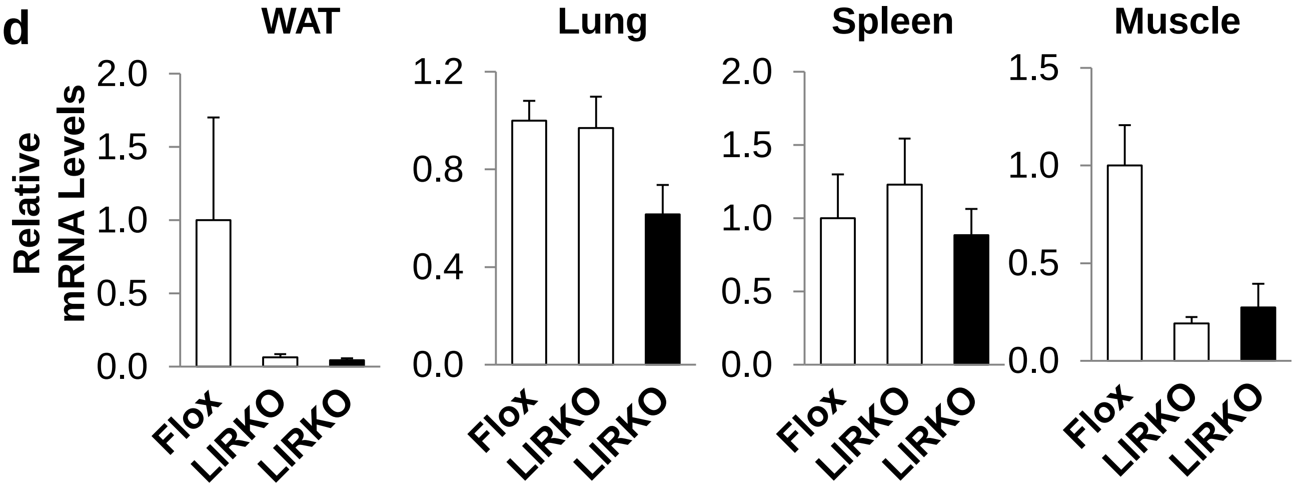

Supplementary Figure 2

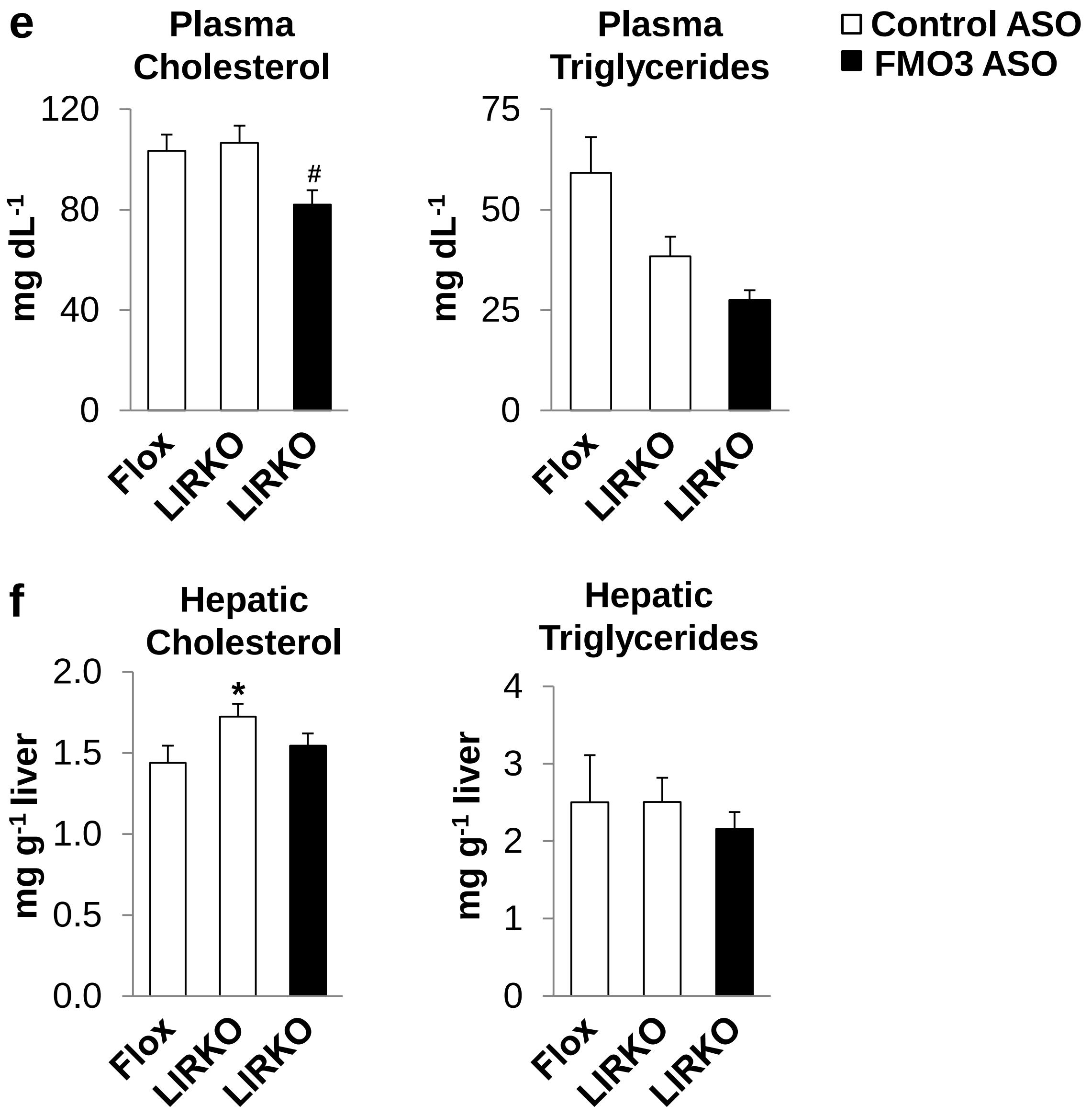

Supplementary Figure 2

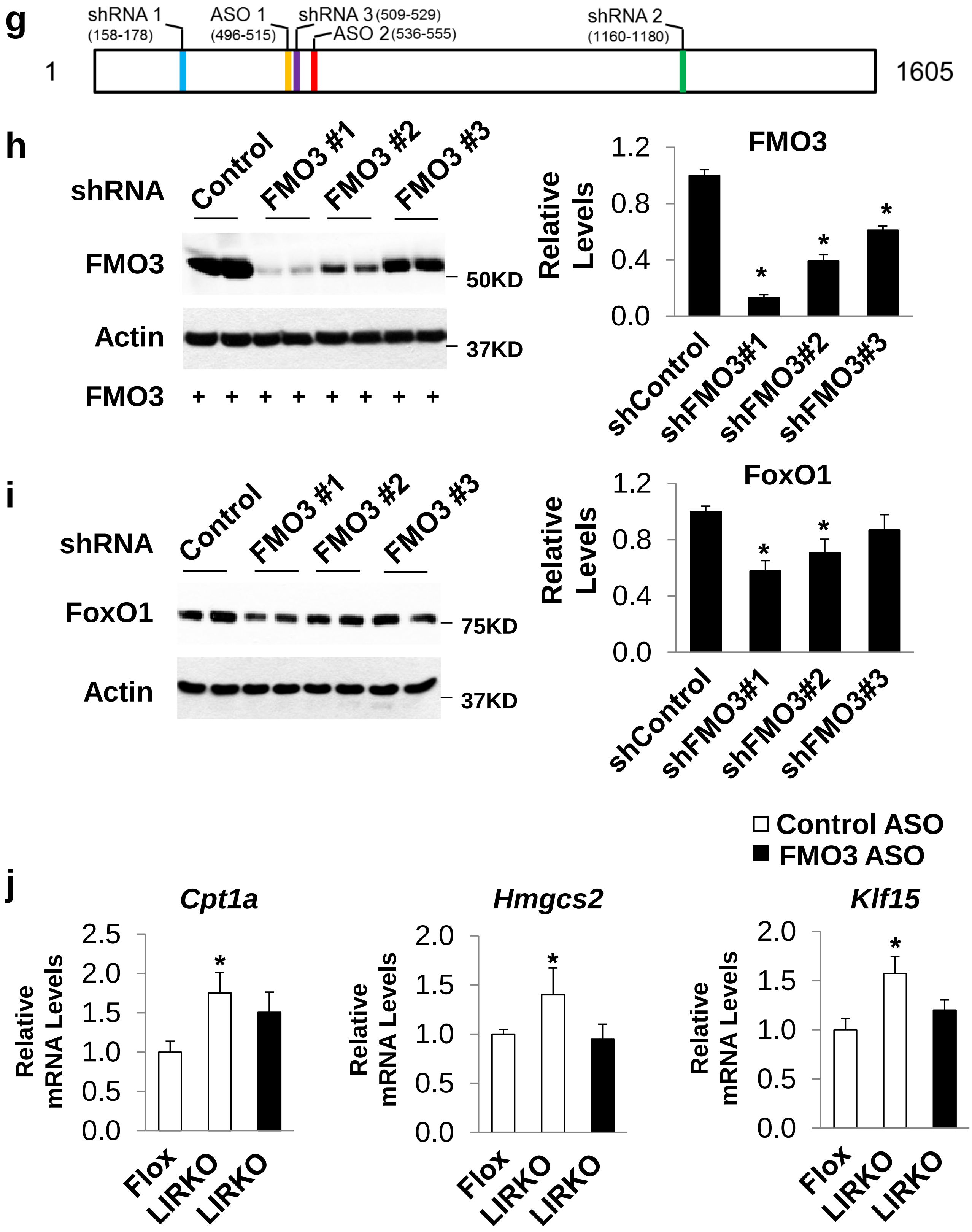

Supplementary Figure 2

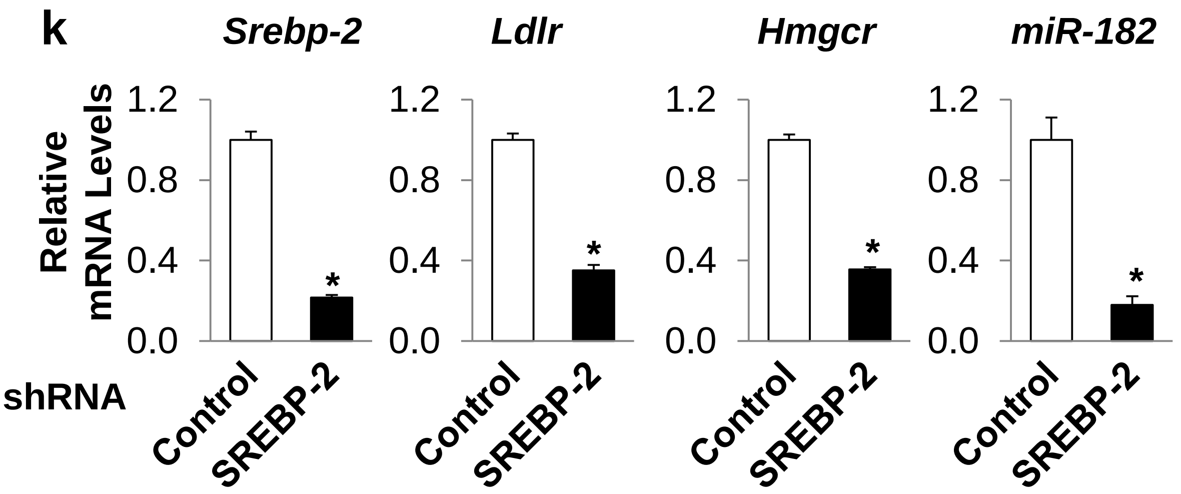

Supplementary Figure 2. Knockdown of FMO3 reduces FoxO1 *in vivo* and *in vitro*.
Four to six week old male Flox and LIRKO mice were treated with control or FMO3 ASO for seven weeks and sacrificed in the non-fasted state, at which point body weight was measured (**a**) and plasma was taken for measuring chemistries (**b**). Gene expression in liver (**c**, **j**) or other tissues (**d**) was measured by real-time PCR. Cholesterol and triglycerides were measured in both plasma drawn after a four-hour fast (**e**) and liver (**f**). Data represent the mean ± SEM; n = 5 - 7; * $p < 0.05$ (Student's t-test), LIRKO versus Flox mice treated with the control ASO; $^{\#}$ $p < 0.05$ control versus FMO3 ASO treated LIRKO mice. (**g**) Schematic of the shRNAs and ASOs against FMO3 used in this study. (h) 293A cells were transfected with wildtype mouse FMO3 and either shlacZ, shFMO3#1, shFMO3#2 or shFMO3#3, to show knockdown of FMO3. A representative western blot shown on left, and quantification of two independent experiments is shown on right. Data represent the mean ± SEM; * $p < 0.05$ (Student's t-test) versus control shRNA transfected wells. (**i**, **k**) H2.35 hepatoma cells were transfected with shRNAs as indicated. (**i**) Whole cell lysates were prepared and subjected to western blotting. Representative western blot shown on left, and quantification of three independent experiments is shown on right. (**k**) Alternatively, gene expression was measured using real-time PCR; representative results of three independent experiments are shown; n = 3 replicates per condition. Data represent the mean ± SEM;. * $p < 0.05$ (Student's t-test) versus control shRNA transfected wells.

Supplementary Figure 3

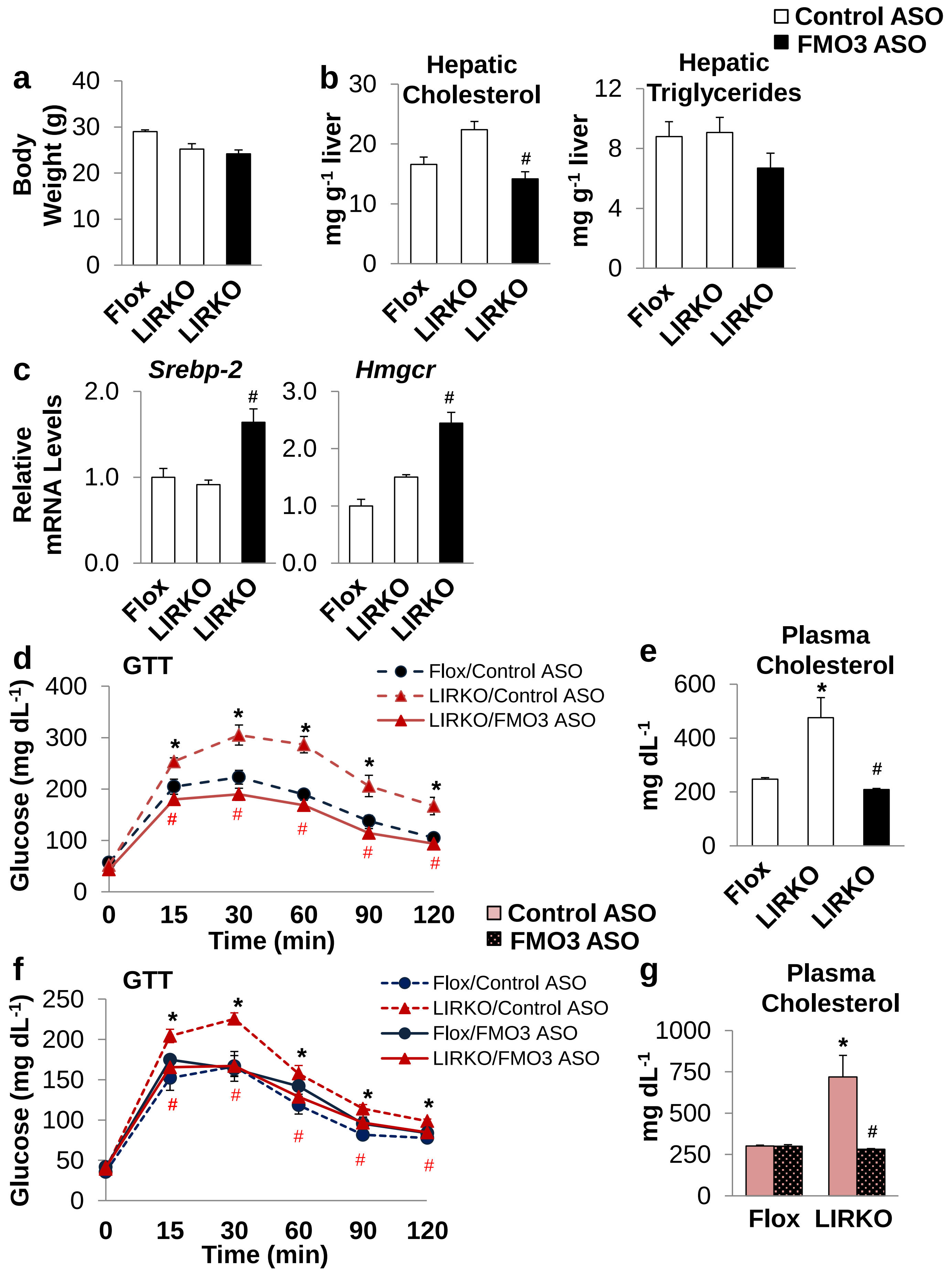

Supplementary Figure 3. Knockdown of FMO3 in LIRKO mice on atherogenic diets.
(**a** to **c**) Four to six week old male Flox and LIRKO mice were placed on an atherogenic Paigen diet and treated with control or FMO3 ASO for 16 weeks. Mice were sacrificed in the non-fasted state, at which time body weights were measured (**a**) and livers collected for measurement of lipids (**b**) and gene expression (**c**). Data represent the mean ± SEM; n = 5 - 7; * $p < 0.05$ (Student's t-test) LIRKO versus Flox mice treated with the control ASO; # $p < 0.05$ (Student's t-test) control versus FMO3 ASO treated LIRKO mice. (**d** to **g**) Four to six week old male (**d**, **e**) and female (**f**, **g**) Flox and LIRKO mice were placed on a Western diet and treated with control or FMO3 ASO for five weeks. Glucose tolerance testing was performed after four doses of ASO (**d**, **f**) and four-hour fasted plasma was collected for cholesterol measurement after five doses of ASO (**e**, **g**). Data represent the mean ± SEM; n = 5; * $p < 0.05$ (Student's t-test) LIRKO versus Flox mice treated with the same ASO; # $p < 0.05$ (Student's t-test) control versus FMO3 ASO treated LIRKO mice.

Supplementary Figure 4

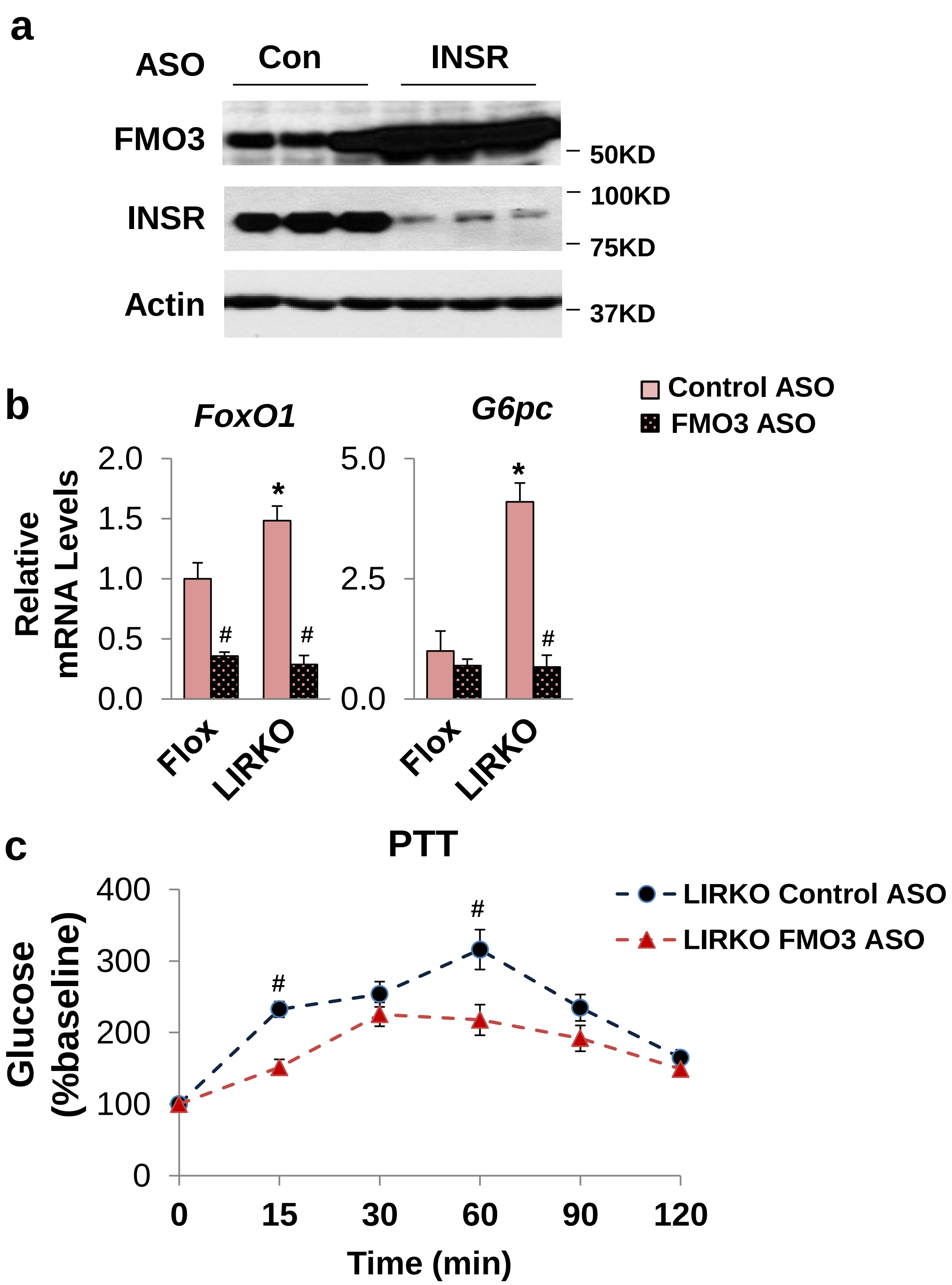

Supplementary Figure 4

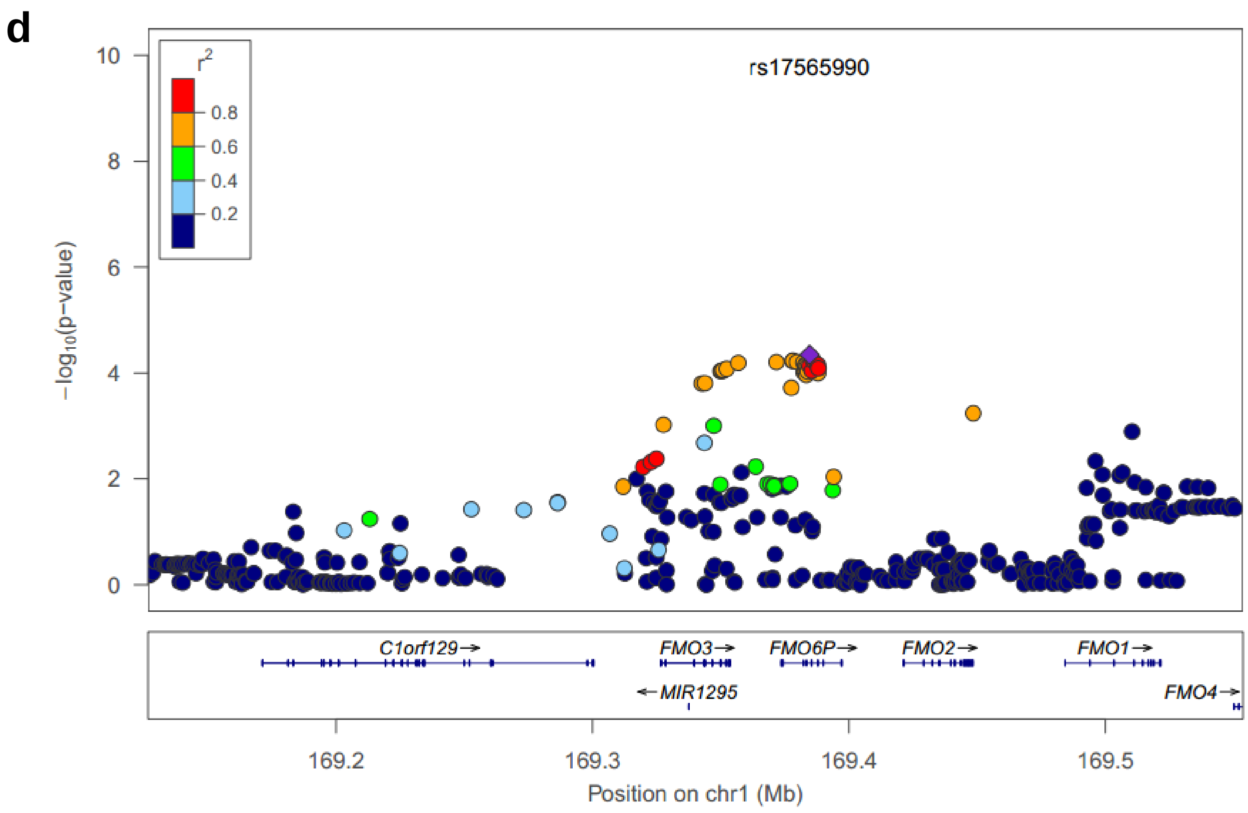

Supplementary Figure 4. FMO3 and glucose metabolism.
(**a**) Six week old male *ob/ob* mice were treated with control ASO (Con) or ASO targeting the insulin receptor (INSR) for four weeks and were sacrificed in the non-fasted state. Protein levels were measured by western blotting whole cell lysates. (**b**, **c**) Four to six week old female Flox and LIRKO mice were treated with control or FMO3 ASO for seven weeks and sacrificed in the non-fasted state. Hepatic gene expression (**b**) was measured by real-time PCR. Pyruvate tolerance testing (**c**) was performed after six weeks of ASO administration. Data represent the mean ± SEM; n = 5; * $p < 0.05$ (Student's t-test) Flox versus similarly treated LIRKO mice; [#] $p < 0.05$ (Student's t-test) control versus FMO3 ASO treated LIRKO mice. (**d**) LocusZoom[1] was used to query the Meta-Analysis of Glucose and Insulin-related traits Consortium (MAGIC) [2] data set (n = 15,234 nondiabetic individuals) for associations between the FMO3 locus and glucose levels. Shown above are associations with glucose levels measured two hours after a glucose challenge and adjusted for body mass index. The purple diamond indicates the lead SNP ($p = 4.51 \times 10^{-5}$). Evaluated SNPs are colored based on their correlation with the lead SNP.

Supplementary Figure 5

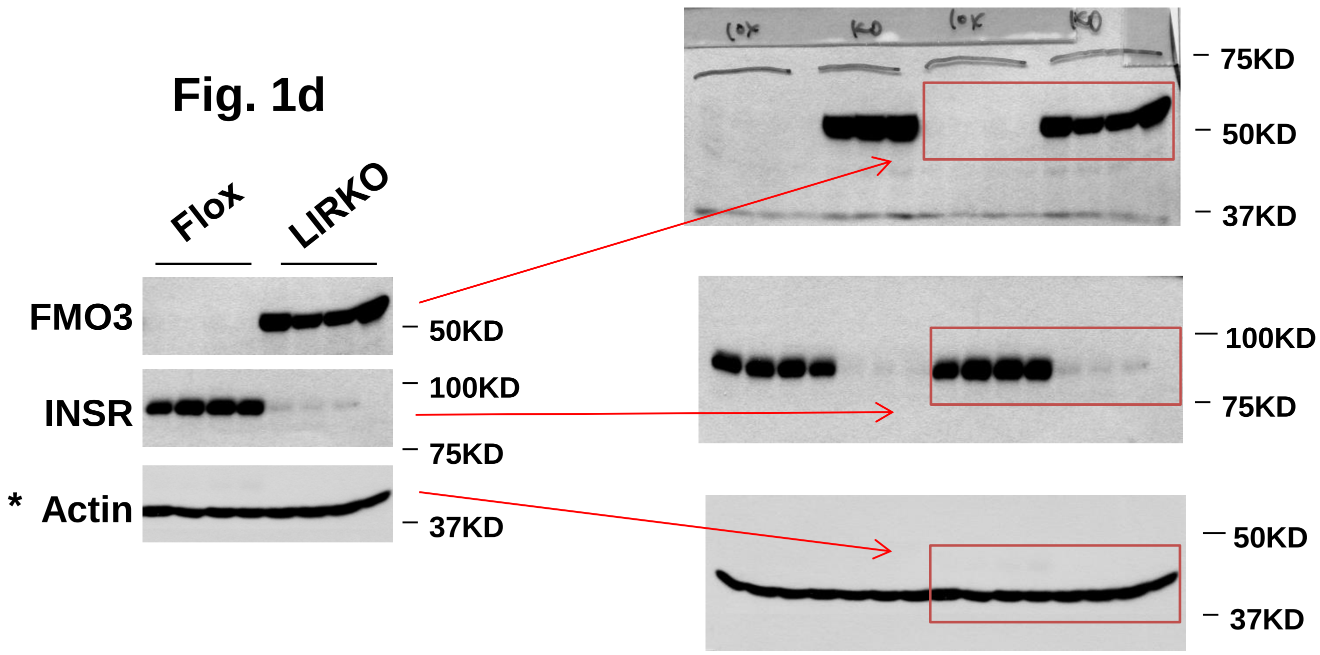

Supplementary Figure 5

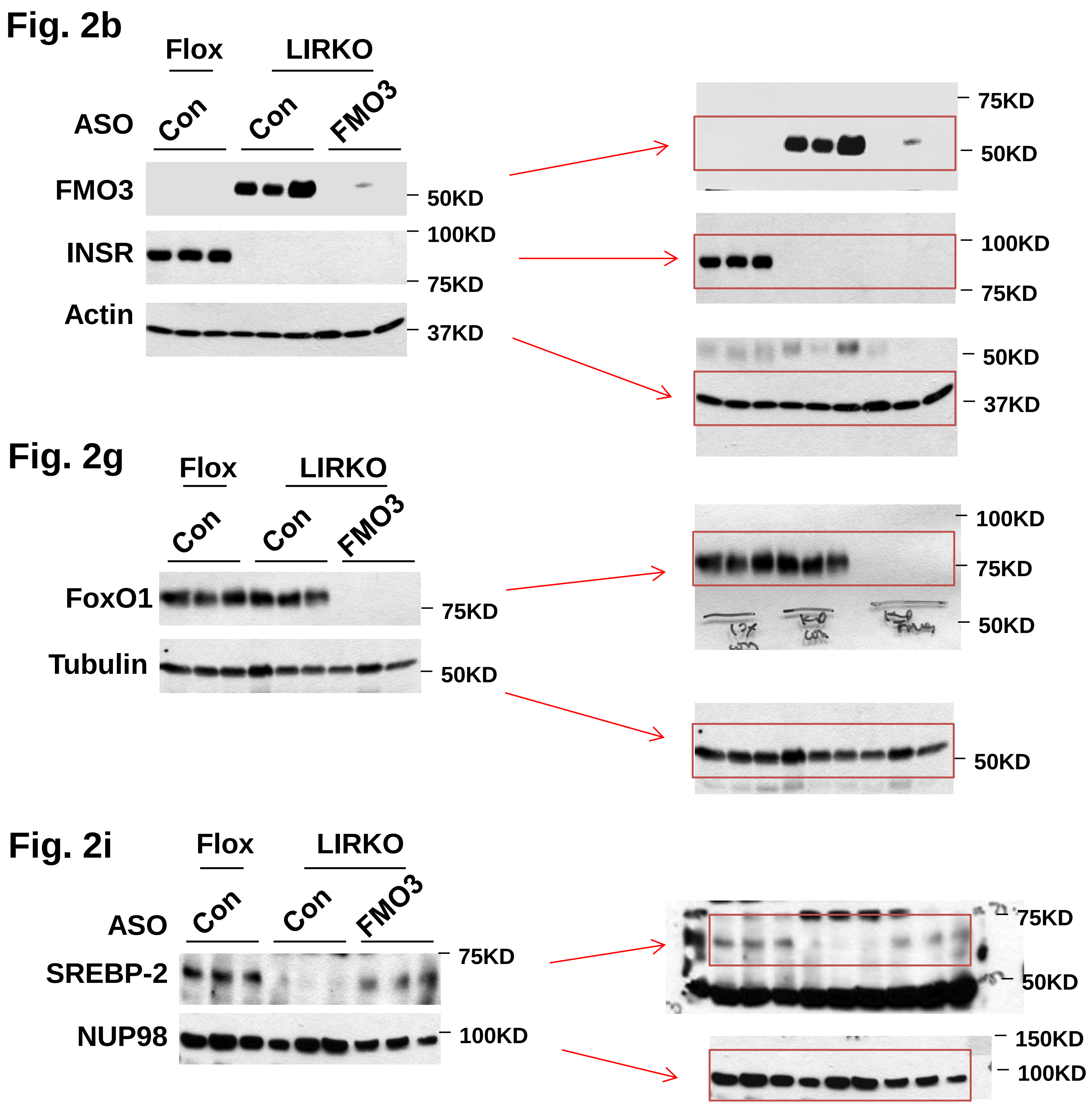

Supplementary Figure 5

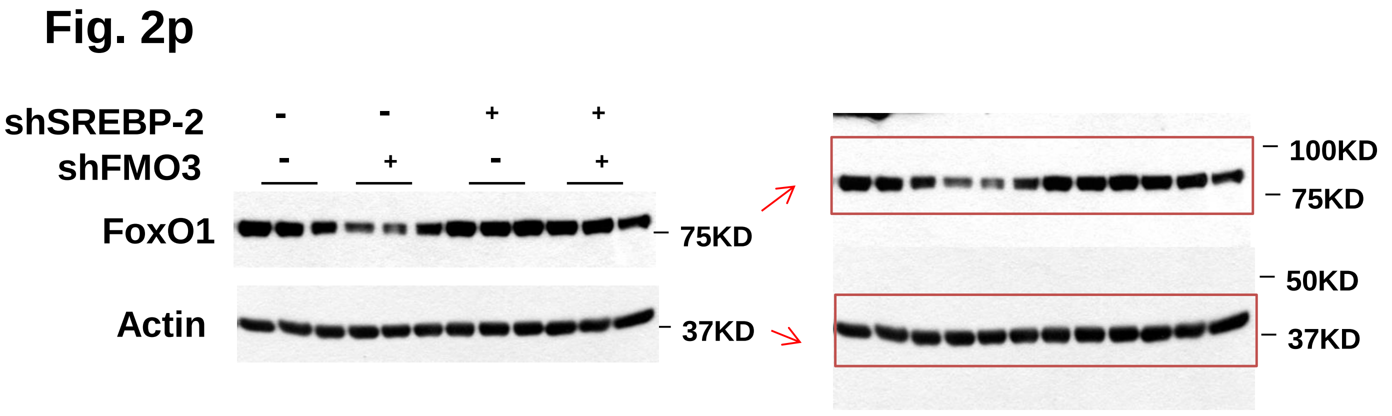

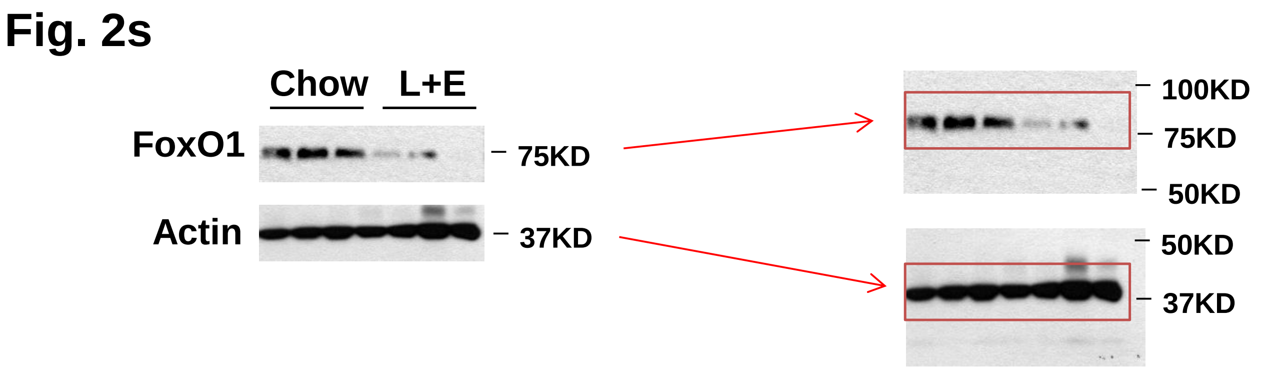

Supplementary Figure 5

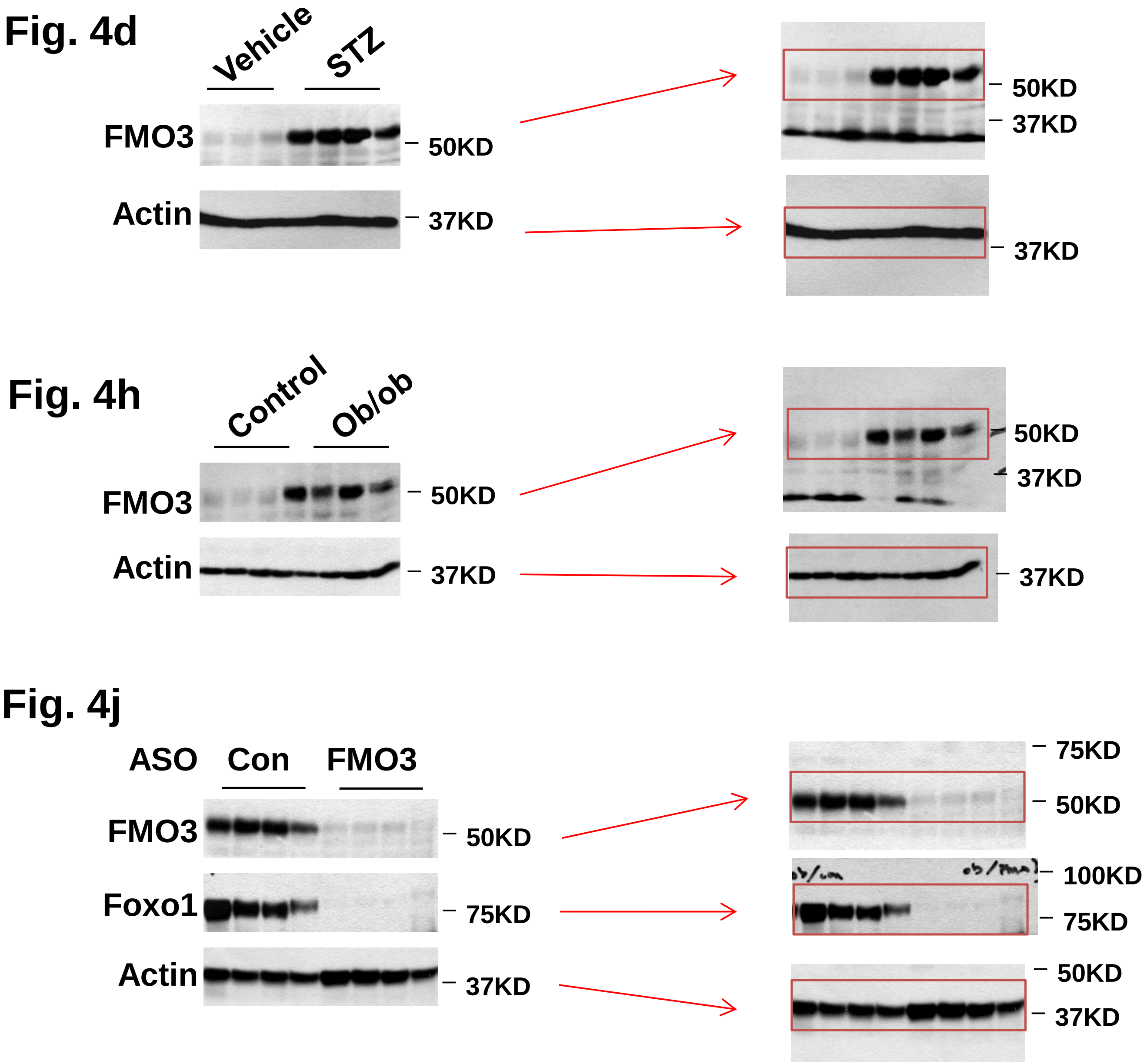

Supplementary Figure 5. Uncropped scans of key western blots.

Supplementary Table 1. Real-time PCR primers used in this study.

Gene	Sequence
Mouse-*Fmo3*-F	GGAACTTGCACTTTGCCTTC
Mouse-*Fmo3*-R	TAGGAGATTGGGCTTTGCAC
Mouse-*Fmo1*-F	AAACAAGCATAGCGGGTTTG
Mouse-*Fmo1*-R	ATCCGGTTTTGCGTTGATAG
Mouse-*Fmo2*-F	AGCTGTGGTCTTCGAGGATG
Mouse-*Fmo2*-R	GGCAAGCTACACAAGCCTTT
Mouse-*Fmo4*-F	CGGAGCAGCTCATTAAAAGG
Mouse-*Fmo4*-R	CTGAGTGAGCTCGTCCATGT
Mouse-*Fmo5*-F	TGCCCTCACAAAGTGAAATG
Mouse-Fmo5-R	GCTGGCTGTCCACATACCTT
Mouse-*FoxO1*-F	TCCCATCATGACAGAGCAGGATGA
Mouse-*FoxO1*-R	CAGACTGGGCAGCGTAGACGC
Mouse-*Pck1*-F	ATCCCAACTCGAGATTCTGC
Mouse-*Pck1*-R	CATGGCTGCTCCTACAAACA
Mouse-*G6pc*-F	TGGCTTTTTCTTTCCTCGAA
Mouse-*G6pc*-R	TCGGAGACTGGTTCAACCTC
Mouse-*Igfbp1*-F	ATTAGCTGCAGCCCAACAGA
Mouse-*Igfbp1*-R	GACACACCAGCAGAGTCCAG
Mouse-*Srebp-2*-F	GCGTTCTGGAGACCATGGA
Mosue-*Srebp-2*-R	ACAAAGTTGCTCTGAAAACAAATCA
Mouse-*Ldlr*-R	GAGGAGCAGCCACATGGTAT
Mouse-*Ldlr*-F	GCTCGTCCTCTGTGGTCTTC
Mouse-*Hmgcr*-F	CTTGTGGAATGCCTTGTGATTG
Mouse-*Hmgcr*-R	AGCCGAAGCAGCACATGAT
Mouse-*Fdps*-R	ATGGAGATGGGCGAGTTCTTC
Mouse-*Fdps*-F	CCGACCTTTCCCGTCACA
Mouse-*Fdft1*-F	CCAACTCAATGGGTCTGTTCCT
Mouse-*Fdft1*-R	TGGCTTAGCAAAGTCTTCCAACT
Mouse-*Cyp51*-F	AGCTGTACGCAGACCTGGAT
Mouse-*Cyp51*-R	ACGCCCGTCCTTGTATGTAG
Mouse-*Srebp-1c*-F	GGCCCGGGAAGTCACTGT
Mouse-*Srebp-1c*-R	GGAGCCATGGATTGCACATT
Mouse-*Fasn*-F	GCTGCGGAAACTTCAGGAAAT
Mouse-*Fasn*-R	AGAGACGTGTCACTCCTGGACTT
Mouse-*Cyp27a1*-F	GGAGGATTGCAGAACTGGAG
Mouse-*Cyp27a1*-R	TGCGGGACACAGTCTTTACTT
Mouse-*Cyp7a1*-F	GTCCGGATATTCAAGGATGCA
Mouse-*Cyp7a1*-R	AGCAACTAAACAACCTGCCAGTACTA
Mouse-*Cyp8b1*-F	GGAACAGCCTATCCTTGGTGA
Mouse-*Cyp8b1*-R	GGCCCCAGTAGGGAGTAGAC
Mouse-*Abca1*-F	CATCCTCTCCCAGAGCAAAA
Mouse-*Abca1*-R	CCACATCCACAACTGTCTGG
Mouse-*Abcg5*-F	TGCCCATTCCTTTAAAAATCC
Mouse-*Abcg5*-R	GATGAACTGGACCCCTTGG

Mouse-*Abcg8*-F	GGGGCTGATGCAGATTCA
Mouse-*Abcg8*-R	GTAGCTGATGCCGATGACAA
Mouse-*Bsep*-F	AAGGACAGCCACACCAACTC
Mouse-*Bsep*-R	CCAGAACATGACAAACGGAA
Mouse-*Oatp1*-F	TAATCGGGCCAATCTTC
Mouse-*Oatp1*-R	ACTCCCATAATGCCCTTGG
Mouse-*Ntcp*-F	TCCGTCGTAGATTCCTTTGC
Mouse-*Ntcp*-R	AGGGGGACATGAACCTCAG
Mouse-*Mrp2*-F	TCTGTGAGTGCAAGAGACAGGT
Mouse-*Mrp2*-R	TCCAGGACCAAGAGATTTGC
Mouse-*Lxr*-F	ACTTCAGTTACAACCGGGAAGA
Mouse-*Lxr*-R	GAGCAAACTCAGCATCATTGAG
Mouse-*Fxr*-F	CACGGTTGTAAATACAGACTAGATAG
Mouse-*Fxr*-R	TTGATTTAATTAGGCCAAAAGG
Mouse-*Shp*-F	CTGCCTGGAGTCTTTCTGGA
Mouse-*Shp*-R	GGTACCAGGGCTCCAAGACT
Mouse-*36b4*-F	AGATTCGGGATATGCTGTTGGC
Mouse-*36b4*-R	TCGGGTCCTAGACCAGTGTTC
Mouse-*18s*-F	GTAACCCGTTGAACCCCATT
Mouse-*18s*-R	CCATCCAATCGGTAGTAGCG
Mouse-*Tat*-F	CACGGCTAGACACAGCTCAA
Mouse-*Tat*-R	CTATCCAGTCGGGAGGAGGT
Rat-*36b4*-F	TTCCCACTGGCTGAAAAGGT
Rat-*36b4*-R	CGCAGCCGCAAATGC
Rat-*Fmo3*-F	GTGTTTTCCAGACTTCCC
Rat-*Fmo3*-R	ATACCACCAGTCAGAAAT
Rat-*Fmo1*-F	GGTCCTGAAAGGTGCGACTA
Rat-*Fmo1*-R	CCAAATCCGCTATGCTTGTT
Rat-*Fmo2*-F	CCTGGTGCCTCAGAACAAAT
Rat-*Fmo2*-R	CTTTGATGGCTCCGTACAGC
Rat-*Fmo4*-F	CAGTGGAATAGCAGCACAGG
Rat-*Fmo4*-R	TTCAAAGGGTAGCCACCAAC
Rat-*Fmo5*-F	TGAAGGTGAAAGGCAATGTG
Rat-*Fmo5*-R	TGTAGCCTGTGGCAAAGATG
Rat-*Pck1*-F	ATGAAGTTTGATGCCCAAGG
Rat-*Pck1*-R	ACCCCCATCACTTGTCTCAG
Rat-*Srebp-1c*-F	GCGCTACCGTTCCTCTATCA
Rat-*Srebp-1c*-R	ACATCTGTGCCTCCTCCACT
Rat-*Tat*-F	AGCTTCCTCAAGTCCAATGC
Rat-*Tat*-R	TTCATCTCAAGTCCAATGC

Supplementary Table 2. C_T values of genes measured by real time PCR.

	Flox or Wildtype Male Mice					**Primary Rat Hepatocytes**
	Liver	WAT	Lung	Spleen	Muscle	
Fmo3	31	28	23	31	28	27
Fmo1	20					26
Fmo2	24					32
Fmo4	25					26
Fmo5	19					23
FoxO1	20					
Pck1	15					25
Igfbp1	21					
Srepb-2	20					
Hmgcr	20					
Fdps	21					
Fdft1	19					
Cyp51	20					
Ldlr	19					
Srebp-1c						24

Supplementary Table 3. Antibodies used in this study.

Protein	Catalog #	Company
FMO3	Ab126790	Abcam
FoxO1	2880	Cell Signaling
NUP98	2598	Cell Signaling
INSR	sc-711	Santa Cruz Biotechnology
SR-B1	NB400-101	Novus Biologicals
Sortilin	Ab16640	Abcam
Beta-actin	sc-47778	Santa Cruz Biotechnology
Alpha-tubulin	sc-8035	Santa Cruz Biotechnology

References

1. Pruim, R.J. et al. LocusZoom: regional visualization of genome-wide association scan results. *Bioinformatics* **26**, 2336-7 (2010).
2. Saxena, R. et al. Genetic variation in GIPR influences the glucose and insulin responses to an oral glucose challenge. *Nat Genet* **42**, 142-8 (2010).
